# Characterizing the influence of racism-related stress and pandemic-related changes in social connections on cardiovascular health: Study protocol and theoretical framework

**DOI:** 10.1371/journal.pone.0324839

**Published:** 2025-07-17

**Authors:** Jewel Scott, Victoria Money, Charles Ellis, Chanita Hughes-Halbert, Michelle A. Birkett, Gayenell Magwood

**Affiliations:** 1 Department of Biobehavioral Health & Nursing Science, University of South Carolina College of Nursing, Columbia, South Carolina, United States of America; 2 Department of Sociology, University of South Carolina, Columbia, South Carolina, United States of America; 3 Department of Speech, Language, and Hearing Sciences, Communication Equity and Outcomes Laboratory, College of Public Health and Health Professions, University of Florida, Gainesville, Florida, United States of America; 4 Department of Population and Public Health Sciences, University of Southern California, Columbia, South Carolina, United States of America; 5 Department of Medical Social Sciences and Center for Computational and Social Sciences in Health (COMPASS), Northwestern University, Evanston, Illinois, United States of America; University of Florida, UNITED STATES OF AMERICA

## Abstract

**Background:**

Cardiovascular-related morbidity and mortality among young adults are rising, and disparities between young Black and White women are immense. Health behaviors including physical activity and diet are essential for optimal cardiovascular health. Psychosocial factors, such as racism, perceived stress, and lack of social support, contribute to racial inequities in cardiovascular disease, which were further exacerbated by the COVID-19 pandemic. The proposed study aims to assess the influence of racism-related stress and other social determinants of health, including social connectedness, on quality of life and cardiovascular-related health behaviors.

**Methods:**

This is a concurrent, triangulation, mixed-methods study combining survey data on health behaviors, psychosocial stressors, and social networks with a thematic analysis of focus group data. Participants will be people who self-identify as Black or African American woman, between the ages of 18 and 39, residing in the southeastern United States with at least one cardiovascular disease risk factor. The primary outcome is physical activity and quality of life.

**Discussion:**

The results will be informative for developing an action plan for improving cardiovascular health at a network and community-level to mitigate the predicted post-pandemic increase in cardiovascular disease. This study will provide an integrated perspective of the influence of the COVID-19 pandemic on quality of life and social relationships with implications for cardiovascular health equity.

## Introduction

Cardiovascular disease (CVD) continues to be the leading cause of death and accounts for nearly 20% of deaths that occur before age 65 in the U.S. [1]. However, CVD onset and diagnosis occur years earlier, on average at 54 years of age for women per data from National Health and Nutrition Examination Surveys [2]. A closer look at the data reveals the mean age of CVD diagnosis for Black women is 6 years earlier than White women (56 years vs 50 years) [2], consistent with the racial disparities in other related outcomes such as stroke [3–5]. In addition, there are regional disparities, with the American South having the highest prevalence of heart disease and stroke in the U.S. [3,6,7].

CVD risk factors, such as hypertension, hyperlipidemia, or obesity, typically develop long before disease onset and death. An analysis of National Inpatient data on young adults (18–59 years of age) experiencing their first myocardial infarction found that 20% of deaths occurred in those aged 18–44, with 90% of these individuals having at least one risk factor [8]. Furthermore, disparities exist both within and across racial groups, with conditions like hypertension, diabetes, and obesity, being more prevalent among Black women compared to Black men and White women [5,8].

Maintaining a healthy diet and engaging in regular physical activity (PA) are key behaviors for reducing CVD risk and promoting optimal cardiovascular health (CVH). However, these behaviors are greatly influenced by the social environment and psychosocial stress. Discrimination, perceived stress, and lack of social support are examples of environmental and psychosocial factors associated with not meeting the recommended guidelines for PA and other CVH behaviors [9–12]. Among Black women, research suggests that chronic psychosocial stress, and related diet-based coping and fatigue, present significant barriers to engaging in sustained health-promoting behaviors [9,10,13]. Understanding these challenges will be critical for developing effective interventions that support lifelong cardiovascular health.

The COVID-19 pandemic was a universal stressor, albeit the intensity of the stress exposure and related consequences varied along racial, economic, and generational lines [14,15]. Many people employed in entry-level and service occupations disproportionately held by young people of color were not able to adjust their work schedule to work from home, contributing to the disproportionate burden of COVID-19 infection among Black young adults. Millennial adults (ages 26–41 in 2022) reported more behavioral-based coping, including increased junk food intake and more sedentary behaviors than older generations [16]. Moreover, today’s youth and young adults are more obese, have more chronic diseases, and report greater psychosocial distress than previous generations [17,18]. For many young Black women, the confluence of pandemic-related stress and social upheaval resulting from the spotlight on racism in America compounded their pandemic-related stress. Among Black women, the most common approach for coping with stress, specifically related to racism, is collective coping—such as seeking support from social connections [19–21]. The COVID-19 pandemic shifted many social relationships and it is unclear whether social connections are rebounding, and what impact these changes may have on CVH.

Social connections, and the support they provide, are important for CVH. Many studies have examined aspects of social relationships, such as social connectedness or social support, and most identify the protective benefits of positive social connections on stress-related biomarkers (e.g., inflammation), hypertension, and health behaviors such as physical activity [22–27]. For example, in the Jackson Heart Study, having social connections that could be counted on for practical assistance (e.g., functional social support) was associated with a lower incidence of hypertension, but the number of connections was not [24]. However, at least one study identified generational differences in the relationship between social connections and distress related to stressful life events [28]. Child and colleagues [28] found that social connections were especially helpful for late-middle aged adults, but not young adults among a sample of California residents. These findings are striking, and merit further investigation in the context of COVID-19.

The COVID-19 pandemic was a once in a lifetime stressor, that was further complicated by the heightened awareness of racial discrimination, illuminating the well-known link between racism and disease conditions. The ubiquity of the pandemic and the floodlight on health inequities and racism, coupled with social distancing protocols to contain the rise in infections, may have frayed social connections or exhausted the reserve capacity of social networks. Thus, the overall objective of the research study is to explore the structural, social, and pandemic-related factors influencing the quality of life and CVH behaviors of young adult Black women. We aim to answer the following two research questions: 1) How does pandemic-related change in social connections relate with current quality of life and CVH Behaviors of young Black women? and 2) What is the relationship between racial trauma, an intermediate structural determinant of health, and health-related quality of life and CVH behaviors among young Black women? The study is guided by frameworks on the impact of social determinants of health on CVD, prevention science, social science and social network theories [29–33].

**Fig 1:**
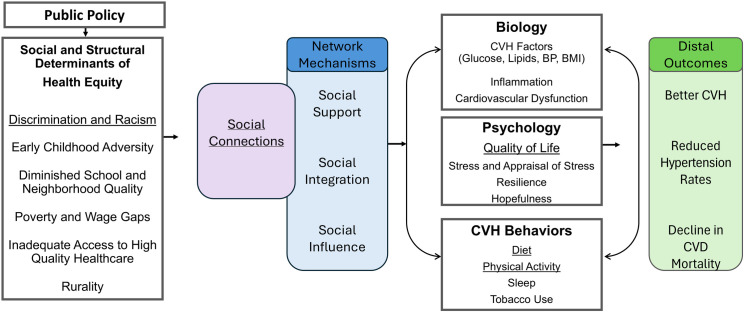
Conceptual framework for the role of social relationships and cardiovascular health equity.

Fig 1 presents a broad conceptual framework derived from several published frameworks and depicts how social connections can be influential for promoting CVH equity for young Black women. The concepts examined in the current study are underlined. Recent work has critically evaluated how structural and social factors that are shaped by the application of laws and policies have a trickle-down effect on the CVH of individuals [29]. Compelling evidence links structural and social determinants of health to cardiovascular and other health outcomes, through physiological processes including inflammation [29,34,35]. Stressful circumstances activate the biological stress-response system, particularly the hypothalamus-pituitary-adrenal (HPA) axis and sympathetic nervous system. While acute stress responses, such as increases in inflammation, are adaptive and protective in the short term (in response to danger or illness), chronic exposure to stressors (such as economic hardship, discrimination, or neighborhood disadvantage) leads to dysregulated HPA axis activity and persistent low-grade inflammation. This prolonged exposure to inflammatory cytokines contributes to endothelial dysfunction, arterial stiffness, and the development of atherosclerosis and hypertension [29,36]. This is one way structural and social determinants become biologically embedded contributing to inequities in CVH [34,35,37]. The examples of structural and social determinants of health in Fig 1 are not exhaustive, but provide illustrations that are especially relevant at the developmental stage of emerging and young adulthood [38].

This framework outlines the hypothesized pathways through which social connectedness and social networks influence cardiovascular health outcomes. Social connectedness operates through key network mechanisms and affects intermediate outcomes, including biological processes, psychological well-being, and health behaviors. These intermediate pathways ultimately contribute to distal cardiovascular health outcomes. CVH = cardiovascular health; CVD = cardiovascular disease

Social connections and the access to resources they provide can be considered a social determinant of health. In fact, the Solar and Irwin model of SDOH that was adapted by the World Health Organization (WHO) situates social connections in a unique position of crossing multiple determinants of health [39]. Social networks enable us to think of social connections in a dynamic way, critically assessing the interdependencies between the network’s structure (e.g., size), the characteristics of individuals within the network, and the dynamics of relationships between individuals or groups that may influence behaviors and related health outcomes. Social connections (e.g., ties) being individuals or groups can act as pathways or barriers to health outcomes, and potentially buffer the detrimental effects of negative social experiences such as poverty or discrimination [30,31,33,40]. Using social networks to measure not only individual behaviors but also the influence of network properties on CVH opens opportunities to critically explore how social connections influence CVH outcomes through various psychosocial mechanisms such as social support, social integration, and social influence [31,33]. Social support, such as emotional or instrumental assistance from others, can buffer stress by reducing perceived burdens and enhancing coping capacity, potentially lowering cardiovascular risk. Social integration, which refers to the extent of participation in a network, promotes a sense of belonging that can mitigate stress and encourage healthy behaviors, such as regular physical activity or adherence to medical advice. Social influence, or the norms and behaviors modeled within a network, can shape lifestyle choices, such as diet or physical activity, which directly impact cardiovascular health [22,41,42].

Lastly, a prevention science lens complements the SDOH framework with an emphasis on the role of policy to effectively address how resources are distributed within a population, structural and social determinants of health including racism and discrimination [32]. This approach integrates a focus on upstream determinants, such as economic and social policies, with strategies to mitigate the downstream effects of structural inequities on CVH, consistent with the WHO SDOH framework.

## Materials and methods

### Study design

**Fig 2:**
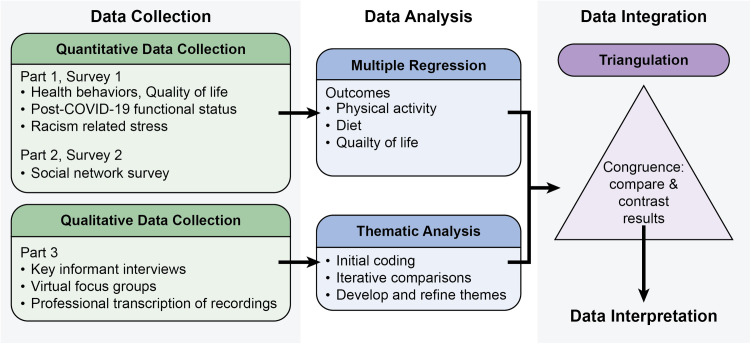
Mixed methods study design.

This is an ongoing, cross-sectional, concurrent triangulation mixed methods study that will provide insight into the status of CVH behaviors of young Black women in the post-peak phase of the COVID-19 pandemic. An advantage of the concurrent triangulation design is the equal weighting of the quantitative and qualitative data (Fig 2) [43]. In part one, participants complete survey measures about medical history, COVID-19 related experiences, health behaviors, and racial stress. In part two, participants complete an online social network survey, and part 3 consists of the collection of qualitative data via key informant interviews and focus groups.

This study uses a concurrent triangulation approach, in which quantitative and qualitative data are collected and analyzed simultaneously to provide a comprehensive understanding of factors influencing physical activity, diet, and quality of life post-COVID-19. Data sources will be weighted equally and integrated during interpretation to validate findings and enhance the depth and credibility of the results.

The University of South Carolina Institutional Review Board reviewed and approved this study. The study was determined to be a minimal risk study and a waiver of written informed consent was obtained. Documentation of consent is provided by the participants reviewing information about the research and clicking a button affirming their intent to participate. They are also provided with the option to request to speak with the research team if they have questions about the study. This process is in line with the Office of Human Research Protections 2015 recommended guidance on minimal risk research and informed consent [44].

### Study sample and recruitment

In collaboration with existing partnerships with community-based organizations, we will recruit 100 women who self-identify as Black or African American. In addition, recruitment will occur through presentations to community and faith-based organizations (e.g., community health centers), Black sororities, and directed advertisements on social media. The inclusion criteria are: 1) self-identify as a Black or African-American woman, 2) between the ages of 18 and 39, 3) history of a diagnosed COVID-19 infection, 4) Speak and understand English, 5) reside in the southeastern U.S. defined as North Carolina, South Carolina, or Georgia, and 5) are at risk of developing CVD [45,46]. To identify the presence of a CVD risk factor we modified the Centers for Disease Control prediabetes risk screening to include sex-specific risks of gestational diabetes and hypertensive disorders of pregnancy [47]. People were excluded if they are currently pregnant because health behaviors such as sleep, or physical activity may be disrupted during pregnancy. Individuals who respond to advertisements complete an online screener to determine eligibility. Screening, consent, and survey data collection are completed using Redcap (Research Electronic Data Capture), a secure, web-based software platform designed to support data capture for research studies [48,49]. Participant recruitment began on March 31, 2024, and is anticipated to be completed by July 1, 2025. Data collection is estimated to be completed by August 30, 2025, and the analysis will be completed by Spring 2026.

### Measures

Participants will complete sociodemographic questionnaires and validated surveys about quality of life, post-COVID-19 functional status, physical activity, diet, and social determinants of health (e.g., experiences of racism) (Table 1). Following this, they will complete an online social network survey to understand how their social relationships have changed since the pandemic and to characterize the social networks young Black women activate to discuss health and important matters, including racial stress.

**Table 1:** Key study measures.

Health Behaviors
Physical Activity – International Physical Activity Questionnaire (IPAQ)
Diet – Mediterranean Eating Pattern for Americans (MEPA)
Quality of Life – PROMIS v1.2 Global Health Scale
**Social Connections**
UCLA Isolation 3-item
Item from Post-Covid-19 Functional Survey
Social Network Survey
Network Size
Network Density
Perceived Change in Communication with Network – Proportion of Network with less communication after the pandemic
Proportion of Network Available to Discuss Health
Network Strain
**Discrimination and Racism**
Index of Racism Related Stress
Racism Related Vigilance
Everyday Discrimination Scale
**Sociodemographic and Clinical Characteristics**
Age
Education Level
Income
Center for Epidemiologic Studies Depression (CES-D) Scale
Promis Short Form -Emotional Distress-Anxiety Scale
Post-COVID-19 functional status (Yorkshire Rehabilitation Scale)

### Primary outcome

Physical activity is the primary outcome due to strong evidence supporting its anti-inflammatory and mood-enhancing properties, which are important for regulating stress and improving CV health. Physical Activity (PA) will be measured with the short-form International Physical Activity Questionnaire (IPAQ-SF) which elicits moderate and vigorous PA over the previous 7 days. The IPAQ is the most widely used subjective measure of PA, including previous use with Black women [50,51].

### Secondary outcomes

Diet and quality of life are the secondary outcomes. Diet will be assessed using the Mediterranean Eating Patterns for Americans (MEPA), a 16-item scale recommended by the American Heart Association (AHA) to assess diet that promotes CVH [52]. Health related quality of life (HRQOL) is a patient-centered metric that correlates with physical and mental health [53], and Black women often have lower HRQOL than women of other racial and ethnic groups [54]. After Covid-19, HRQOL also varies based on history of infection, severity of infection, gender, and comorbidities [55,56]. We measure HRQOL using the 10-item PROMIS Global Health scale [53].

### Exposures

We assess pandemic-related changes in social connections via a single item from the COVID-19 Yorkshire Rehabilitation Scale that asks participants to rate their problems with socializing and interacting with friends before COVID-19 and post-COVID-19 using a 4-point Likert scale [57]. Social connections are also measured using data from tjhe rigorous social network survey. Three name generators were used to compile personal networks for each participant: 1. Who are the people in your life with whom you discuss important matters?, 2. Name the people with whom you socialize routinely, and 3. Who do you talk to about your physical or mental health?. For each person named, they indicate how often they are in communication, whether it has changed since the COVID-19 pandemic, and whether each person named is connected to each other. Table 1 includes the social network metrics that will be evaluated for their relationship with quality of life and CVH behaviors. Structural characteristics, such as network size and network density, are commonly used in networks and health literature to provide information about the possible flow of resources and information [22,33,41,58,59]. Perceived change in communication – more, same, less, is available for each network member. We will examine the proportion of the network with less communication post-pandemic. Functional characteristics of networks also shape health behaviors, such as providing support for behavioral change. We will explore discussions of health within the network and perceived hassles or strain as potential facilitators or barriers to engaging in physical activity and consuming a heart healthy diet.

Racism will be measured using the Everyday Discrimination Scale adjusted to ask specifically about how often they encountered the list of experiences due to their race or ethnicity [60]. Participants indicate if they experienced it, and the frequency of the discrimination. To capture traumatic aspects of racism, we include a 6-item measure of racism-related vigilance derived from the Chicago Health study which has been used by other researchers [61,62].

To measure racial stress, the Index of Race-Related Stress (IRRS)-Brief Version was adapted for use in a social network survey [63]. The IRRS asks about exposure to racial stress across three dimensions; interpersonal, institutional, and cultural racism. Participants indicate whether they have experienced a specific stress and their reaction to the experience on a 4-item Likert scale ranging from *it did not bother me (0) to being extremely upset* (3). Next, participants indicate if the stressor was discussed with each person named (i.e., alter). To our knowledge, social network analysis has not previously been used to examine discussion of racial stress within networks, thus our findings will provide insights into this novel use of social network methods.

Data on sociodemographic and clinical characteristics will be collected to be included as covariates in statistical modeling given their potential influence on physical activity, diet, and quality of life. Age, education level and income are key sociodemographic characteristics, though education and income may not be modeled together due to their high correlation and limited statistical power. Clinical characteristics such as post-COVID-19 functional status (Yorkshire Rehabilitation Scale), comorbid conditions (e.g., hypertension, diabetes), and mental health status (CES-D and Promis Short Form -Emotional Distress-Anxiety Scale) will also be considered as they may impact engagement in health behaviors and overall well-being.

### Quantitative methods and analysis

We will analyze the independent impact of social drivers of health on total weekly minutes of moderate-vigorous physical activity using separate multiple regression models. A forward hierarchical regression approach will be used. The initial block of predictors (model 1) will be the social determinant of health (e.g., pandemic-related changes in social connections, racism, and racial stress) and community-level SSDH (e.g., social deprivation index and concentrated poverty). The second block of predictors (model 2) will add individual sociodemographic factors such as age, income, and post-COVID-19 functional status because of their relationship with the outcome of physical activity. The forward hierarchical approach will be repeated for the secondary outcomes, diet and quality of life. In addition, the goodness of fit of the models will be examined by calculating the adjusted R-squares. We will also investigate the potential nonlinear relationship between the outcomes and the predictors by applying the polynomial regression models. Data management and visualizations of networks will be conducted using R. For network descriptive statistics and visualizations, the igraph and ideanet packages in R will be used.

### Sample size and power calculation

We estimate the number of participants to detect a 10–15-minute/week change in physical activity assessed with the IPAQ. Any increase in physical activity has cardiovascular benefits, but the goal of increasing physical activity by 5–10% of the recommended 150 minutes/week is the standard recommendation [64,65]. Anticipating a medium effect size (f2 ≈ 0.15) and 5–8 independent variables, 55 participants are needed for regression analyses. As the recruitment target is 100 total participants and to retain a minimum of 65% of participants for the social network survey (n = 66), thus we will be adequately powered for the primary analyses.

### Qualitative methods and analysis

Key informant interviews and focus groups will be conducted concurrently with the quantitative data collection to collect contextual data on the impact of the COVID-19 pandemic and stressors on health behaviors and quality of life. Key informant interviews (N = 3–5) will be used to develop and refine the focus group interview guide. Based on the population and nature of the inquiry, we estimate thematic saturation will be achieved in three-four focus groups of 6–8 individuals each. The groups will be conducted using secure videoconference technology to allow the participant maximum flexibility to complete the focus group from their preferred location; reducing the participant burden related to travel. A video connection permits face-to-face communication which facilitates rapport building, essential for discussing potentially sensitive topics, such as racism. Audio recordings will be professionally transcribed.

Thematic analysis of the qualitative data will be used to identify patterns and extract commonalities from the data that are then grouped into themes. We will use inductive coding to derive the themes from the data, consistent with research focused on lived experiences as opposed to a researcher-driven critique. Transcripts will be coded by two independent coders, who will develop independent coding schemes iteratively using qualitative analysis software. Final coding schemes will be defined in collaboration with the investigator team, who will also adjudicate discrepancies.

### Data integration

To triangulate our findings, we will compare and contrast the themes from the qualitative analysis and the significant findings from the quantitative analysis to identify areas of congruence and contrast. Findings will be represented visually using a joint display technique. Table 2 summarizes the quantitative and qualitative data available to answer each research question.

**Table 2:** Research questions and available data.

Research Question	Related Quantitative data	Related Qualitative data
How does pandemic-related change in social connections relate with current HRQOL and CVH Behaviors of young Black women?	Exposures: 1.Change from pre-COVID and now – Problems with socializing/interacting with friends* or caring for dependents *related to your illness and not due to social distancing/lockdown measures – Covid-19 Yorkshire Rehabilitation Scale 2.Social Network Survey Network size and Change in communication with network members since the pandemic Outcomes: 1.IPAQ Physical Activity 2.MEPA Diet 3.Health Related Quality of Life – PROMIS Global Health Measure	What comes to your mind when you think about how the Covid pandemic affected young Black women’s quality of life? We are now several years from when the pandemic started in 2020. How are you feeling about your social connections now? Do you think the social connections are important for heart health?
What is the relationship between racial trauma, an intermediate structural determinant of health and health-related quality of life and CVH behaviors among young Black women?	Exposures: 1.Everyday Discrimination Scale 2.Index of Race-Related Stress 3.Social Network Survey – percentage of network engaged in discussions about racism related stress *Outcomes*: 1.IPAQ Physical Activity 2.MEPA Diet 3.Health Related Quality of Life – PROMIS Global Health Measure	Overlapping with the COVID-19 pandemic, many things happened to bring discussions about racism to the forefront. How has that impacted you personally? What helps you to manage any feelings or emotions related to racism?

HRQOL = Health Related Quality of Life, CVH = Cardiovascular Health; IPAQ = International Physical Activity Questionnaire, MEPA = Mediterranean Eating Pattern for Americans.

## Results

Final IRB approval was obtained in Spring 2024, and we began enrollment in March 2024. As of Fall 2024, we have enrolled 48 participants, and 39 have completed the study. Enrollment is ongoing until the targeted number is reached.

## Discussion

While impacts from the COVID-19 pandemic were felt around the world, the future impact on health outcomes may not affect all populations uniformly. The current study aims to understand the impact of the pandemic on young Black women given their history of excessive CVD risk above and beyond their peers, which may have worsened during the pandemic, coupled with stressors related to racism and other forms of discrimination. Further, COVID-19 infections, even mild (e.g., nonhospitalized) cases, are associated with declines in functioning (e.g., fatigue, shortness of breath, stress) that could hinder health behavior change and strain social support systems [66–68].

## Study implications

This innovative study focuses on cardiovascular health in young adulthood in a population at high risk of poor CVH and explores the potential for social connections to mitigate pandemic-related impacts on CVH. Completion of the study will provide new insights into the complexities of social relationships through the rigorous assessment of social networks. This is among the first studies to use social network analysis to explore the potential of social connections to mitigate the variety of stressors experienced by young adult Black women, including racial stress. The use of multiple methodologies will facilitate a deeper understanding of the opportunities to leverage the strengths of social relationships to address chronic disparities in CVD.

A rising trend in CVD mortality in the young adult population prompted multiple agencies (e.g., National Institutes of Health, American Academy of Nursing) to publish statements identifying the need to increase research that focuses on CVD prevention in young women [46,69,70]. Our focus on young Black women is responsive to these startling trends in CVD mortality. Moreover, the U.S. Surgeon General’s 2023 report on the epidemic of loneliness and isolation reports on the association of social connections with cardiovascular outcomes and acknowledges the gap in knowledge related to longer term consequences among young adults [71].

## Strengths and limitations

This study asks about pandemic related changes in sleep, functional status, and social connection retrospectively. Thus, there is the risk of recall bias, and to a lesser extent, social desirability bias. In addition, the study includes a single assessment of social networks and racial stress; thus, the analysis is limited to a cross-sectional snapshot of potential relationships between social connections and CVH. Relatedly, causality cannot be inferred from the cross-sectional data but should be a priority for future research investigating the impact of social connections at this critical developmental period on future CVH. In addition, the current study is restricted to young adult Black women in a region of the southeastern U.S., an area with a history notable for its strong connections with the transatlantic slave trade, Jim Crow South, and contemporary challenges with racial tensions and stress. While the findings are not generalizable to young adult Black women across the country, the experiences of young Black women in this region may provide insights into future research considerations for young Black women in the American South, a region plagued by a high prevalence of CVD, and potentially across the U.S., as racial stress is not limited to the Southeast.

The study includes a rigorous assessment of the social networks of young adult Black women, addressing a significant gap in the existing literature. Upon completion, the study will be one of the few to assess contemporary stressors, including racial stress, in relation to social networks using quantitative methods among young Black women. Moreover, the focus on a population that has chronically high rates of hypertension, CVD, and risk of stroke, but is under-represented in the literature on social relationships and health is a significant strength of the research protocol. Lastly, the mixed methods approach supports the synthesis of quantitative findings alongside lived experience and may identify areas of congruence as well as inconsistencies and gaps that can inform further observational and interventional research. Social networks exert their influence on health over time, so priorities for future research will include longitudinal follow-up to monitor how changes in social networks, especially during life transitions such as parenting, shape the cardiovascular health of young women. We look forward to opportunities to fund and collect such data, and to explore innovative analytical approaches including dyadic regression analyses or egocentric exponential random graph models (ERGMs) that do not require longitudinal data collection [72].

### Plans for dissemination

Results of the study will be circulated via community forums, research conferences, and peer-review publications. In the future, a community advisory board will be established to further guide the research and to provide input on community-based avenues for research dissemination. In addition, the findings will also serve as pilot data for extramural funding to develop the proposed solutions.
